# Endocrine Outcome and Quality of Life After Transsphenoidal Resection of Pituitary Adenoma—A Prospective Randomized Single-Blinded Study Comparing Endoscopic Versus Microscopic Resection

**DOI:** 10.3390/neurolint17010005

**Published:** 2025-01-10

**Authors:** Andrej Pala, Nadja Grübel, Benjamin Mayer, Ralf Becker, Fabian Sommer, Bernd Schmitz, Gwendolin Etzrodt-Walter, Christian Rainer Wirtz, Michal Hlavac

**Affiliations:** 1 Department of Neurosurgery, University of Ulm, Lindenallee 2, 89312 Günzburg, Germany; nc.grueben@gmail.com (N.G.); rainer.wirtz@bkh-guenzburg.de (C.R.W.); michal.hlavac@uni-ulm.de (M.H.); 2 Institute of Epidemiology and Medical Biometry, University of Ulm, Schwabstr. 13, 89075 Ulm, Germany; benjamin.mayer@uni-ulm.de; 3 Department of Otolaryngology-Head and Neck Surgery, University of Ulm, Frauensteige 12, 89075 Ulm, Germany; 4 Department of Neuroradiology, University of Ulm, Lindenallee 2, 89312 Günzburg, Germany; bernd.schmitz@uni-ulm.de; 5 Endokrinologiezentrum Ulm, Weinbergweg 41, 89075 Ulm, Germany; etzrodtwalter@gmail.com

**Keywords:** transsphenoidal pituitary surgery, hypopituitarism, arginine vasopressin deficiency, endoscopic technique, microscopic technique

## Abstract

Background: Endoscopic pituitary surgery might yield better endocrine outcomes compared to microscopic resection. We conducted a prospective, randomized, single-blinded study to compare the endocrine outcome and quality of life (QoL) of patients with newly diagnosed pituitary adenoma who underwent either endoscopic or microscopic transsphenoidal surgery (NCT03515603). Methods: Due to slow recruitment, this study had to be stopped prematurely. Out of 170 transsphenoidal pituitary surgeries performed during the study period, 36 patients were enrolled in this study. The primary endpoint was based on the development of a new hypopituitarism. Secondary endpoints included the extent of resection, complications, and QoL. Results: Endoscopic surgery was performed in 47.2% (n = 17). A new hypopituitarism was found in 8.3% (n = 3). All these cases underwent microscopic resection. Arginine vasopressin deficiency was found in 2.7% (n = 1) after microscopic resection. Gross total resection was achieved in 94.4% (n = 34). No surgical complications or new neurological deficits were observed. QoL improved significantly after the surgery, as measured by EQ-VAS (*p* = 0.003). According to EQ-5D3L, QoL improved or remained unchanged in almost all patients. No significant difference was found in QoL between the endoscopic and microscopic groups. Conclusion: The endoscopic technique appears to offer benefits in the treatment of pituitary adenomas, particularly in terms of achieving a favorable endocrine outcome.

## 1. Introduction

Pituitary adenomas are the most prevalent tumors in the sellar region, comprising both functional and non-functional neoplasms [1,2]. While prolactinomas follow a different treatment path, transsphenoidal surgery emerges as the primary intervention for most pituitary adenomas, with the critical objective of achieving maximal safe tumor resection [2,3,4]. Historically, neurosurgeons relied on microscopic techniques that offered a stereoscopic surgical view. This traditional approach, familiar to surgeons, could be performed by a single surgeon. However, it suffered from significant limitations, including suboptimal illumination and a constrained field of view through a long and narrow surgical corridor [5,6].

Since the introduction of endoscopic techniques in pituitary surgery, the method has gained widespread acceptance, leading to ongoing advancements in transsphenoidal surgery. Endoscopic methods provide compelling advantages: a panoramic view that enables comprehensive exposure of surrounding structures, superior illumination, and enhanced magnification. This approach does present a challenge—it requires two surgeons working in close coordination, each mastering a new and complex surgical technique [7,8,9].

However, there is currently no prospective randomized trial that has shown a clear advantage of one surgical technique over the other. The prospective Transspher study, as well as some earlier retrospective studies, have demonstrated the potential benefit of the endoscopic technique in terms of endocrine outcome [10,11]. Similar trends were observed in our earlier studies [11].

One of the key advantage of the endoscopic approach is the enhanced visualization of the pituitary gland. Improved imaging allows for better tumor delineation and facilitates a more precise preparation of the adenoma while minimizing manipulation and trauma to the surrounding glandular tissue [6]. Furthermore, the improvement in or preservation of a good quality of life is another important goal of surgery for benign adenomas, especially considering the long life expectancy of affected patients.

Based on these findings, we have initiated a prospective, randomized, single-blinded study to compare endocrine outcomes between endoscopic and microscopic resection. The secondary outcome parameters of extent of resection (EoR), QoL, and complications were also analyzed.

## 2. Materials and Methods

Ethics and Informed Consent:

This study was conducted with strict adherence to the ethical principles outlined in the Declaration of Helsinki. Ethical approval was obtained from the local ethics committee (Ethikkommission der Universität Ulm, Ulm, Germany) under approval number 451/17, dated 7 March 2018. The clinical trial was registered with ClinicalTrials.gov, identifier NCT03515603. Prior to participation, all patients provided written informed consent.

Patients and follow-up:

A prospective, randomized, single-blinded, and single-center study comparing endocrine outcomes after either endoscopic or microscopic transsphenoidal surgery was conducted at our department between May 2018 and March 2022. Unfortunately, due to slow patient recruitment, this study had to be terminated prematurely as most patients preferred endoscopic resection.

Out of 170 transsphenoidal pituitary surgeries conducted during the study period, 36 patients were enrolled in this study. All patients with newly suspected pituitary adenomas selected for surgical therapy older than 18 years with signed informed consent were eligible. The primary study endpoint was to evaluate the endocrine outcome 3–6 months after surgery. The endocrinologist, radiologist, and patients were blinded for type of surgical resection. Endocrine outcome was categorized as good if no new deficit developed and bad if a new hypopituitarism for one or more pituitary axes developed after surgery. Pituitary adenomas were graded according to criteria published by Knosp et al. [12]. Adenomas with Knosp grades 0–2 were considered less invasive, while adenomas with Knosp grades 3–4 were considered invasive. Since endocrine function and not the EoR was the primary endpoint, further subdivision of the Knosp grades was deemed not necessary. EoR, QoL according to EQ-VAS, EQ-5D3L, and complications were evaluated as secondary endpoints. Patients were matched according to Knosp grades 0–2 or 3–4. A stratified 1:1 randomization in Knosp subgroups was carried out by the Institute for Epidemiology and Medical Biometry of Ulm University.

Postoperative assessment of clinical and endocrine status as well as MRI were performed 3–4 and 12–15 months after surgery. Thereafter, serial MRI scanning was performed on a yearly basis. PFS was defined as a new suspected tumor lesion in follow-up MRI or increase in postoperative tumor remnant in case of subtotal resection. In hormone-producing tumors, recurrence of hormone excess was defined as progression. Basic demographic data (age, sex) as well as basic adenoma characteristics (volume, subtype of adenoma) were recorded.

Pituitary function evaluation was conducted in collaboration with endocrinologists as part of a multidisciplinary approach. This evaluation occurred before surgery, 4–6 weeks after surgery, and again 3–6 months after surgery. The assessment included measuring baseline levels of pituitary hormones and conducting hypoglycemic testing to assess cortisol and GH dynamics. In cases where hypoglycemic testing was contraindicated, a CRH-test was performed instead. For statistical analysis, new hypopituitarism was defined as a worsening of endocrine function in one or more pituitary axes, or the development of new permanent arginine vasopressin deficiency after surgery.

In patients with acromegaly, disease remission was defined as having a normal IGF-1 level along with either a suppressed GH level of less than 0.4 ng/mL during an oral glucose tolerance test or a GH level of less than 1.0 ng/mL in random sampling. For patients with Cushing’s disease, remission was defined as either the need for cortisol substitution or having a morning cortisol level within the normal range, accompanied by positive suppression to below 2 µg/dl after low-dose dexamethasone exposure.

Additionally, any visual deficits caused by tumor-induced compression of the optic apparatus were documented prior to surgery and monitored during follow-up. The changes in visual alterations were evaluated to determine if there was improvement, stability, or worsening of the visual deficits over time.

OR Setup and MRI:

At our department, we have access to an intraoperative 1.5 T MRI Espree scanner, provided by Siemens AG, Erlangen, Germany. This scanner serves as a one-room solution for intraoperative imaging. During the surgery, analysis of residual tumor was performed using Brainlab Elements software, which allowed for the semiautomatic analysis of thin slice (2 mm) high-resolution coronal and sagittal T2 and contrast-enhanced T1 images. Postoperative MRI scans were performed at 3–4 months and 12–15 months after the surgery. These scans were acquired either using the intraoperative scanner or the 1.5 T MRI Symphony system, also from Siemens AG. Performing the intraoperative MRI (iMRI) scans, including all the mentioned sequences, generally added an additional surgical time of 45 min. It is important to note that all patients included in this study underwent iMRI examination.

MRI volumetric assessment:

The preoperative MRI images included coronal T2-weighted turbo spin echo sequences, as well as coronal and sagittal T1 plain and contrast-enhanced sequences. After image fusion using the aforementioned software, the tumor volume was measured. The tumor borders were segmented semiautomatically on the coronal and sagittal T2-weighted and gadolinium-enhanced T1 images. For the volumetric resection analysis, the preoperative, intraoperative, and postoperative MRI data acquired at 3 months after surgery were used. In cases where tumor borders were unclear, additional MRI images taken 1 year after the surgery were utilized for evaluation. Gross total resection (GTR) was presumed if the postoperative MRI showed no suspicious remnants. EoR was calculated based on the intraoperative MRI (iMRI) as well as the postoperative MRI taken 3 months after surgery. The MRI assessments and volumetric analysis were performed in collaboration with the department of neuroradiology. All MRI scans were reviewed by a radiologist and an experienced neurosurgeon. An experienced radiologist is always available for the evaluation of iMRI images.

Surgical procedure:

The endoscopic transsphenoidal approach was carried out by two highly experienced neurosurgeons, M.H. and A.P., who have extensive experience in both endoscopic and microscopic transsphenoidal surgeries. A binostril approach was used, utilizing rigid 0°, 30°, and 45° Hopkins endoscopes with a 4 hands technique for all endoscopic cases. A turbinectomy was not necessary in any of the included cases. For the microscopic approach, the same experienced neurosurgeons utilized a direct unilateral transnasal paraseptal route. Both the endoscopic and microscopic procedures employed a navigation system from Brainlab, Munich, Germany. In cases where there was minimal or no intraoperative cerebrospinal fluid (CSF) leak, the sella (sella turcica) reconstruction was performed using a fibrin-coated sponge. However, for larger defects, a multilayer technique was employed using abdominal subcutaneous fat graft and fibrin-coated sponge to seal the defect [13].

The surgical complications that were evaluated included the occurrence of a cerebrospinal fluid (CSF) fistula, which was considered a complication if the patient required a lumbar drain or surgical revision due to rhinoliquorrhea (leakage of CSF from the nose) after the transsphenoidal resection. Criteria for meningitis were defined as a positive CSF culture or the initiation of antibiotic treatment for clinical signs of meningitis, even in the absence of a positive culture. Additionally, any new transient or permanent neurological deficit, thromboembolic complications, epistaxis, or any other postoperative bleedings were evaluated as potential complications.

Quality of life assessment:

Standardized questionnaires, including EQ-VAS (EuroQol Visual Analog Scale), EQ-5D3L (EuroQol 5 Dimensions 3 Levels), and Sino-Nasal Outcome Test-20 German adopted version (SNOT-20 GAV), were administered to patients both before surgery and during the first follow-up. The EQ-VAS measures an individual’s self-rated health on a visual scale, while the EQ-5D3L assesses health-related quality of life across multiple dimensions, such as mobility, self-care, usual activities, pain/discomfort, and anxiety/depression. The SNOT-20 GAV questionnaire specifically evaluates nasal symptoms and the impact on daily functioning and quality of life [14].

Data analysis:

Sample size for this study was estimated on retrospective data, suggesting a total number of approximately 100 patients to reach a power of 80% assuming a two-sided error level of 5%. Statistical analysis was performed using SPSS 26.0 (Lead Technologies, INC, Charlotte, NC, USA). Descriptive statistics as well as Chi square, Fisher exact test and Mann–Whitney-U test were used. Because of the low number of patients, multivariable analysis was excluded, and all analysis are interpreted in an explorative manner only. The significance level was set as *p* < 0.05. Original data can be accessed upon request by contacting the corresponding author.

## 3. Results

General characteristics:

Most common were female patients and non-functioning adenomas graded as Knosp 0–2 (Table 1). Median follow-up was 18 months. Median tumor volume was 3.34 cm^3^. Endoscopic resection was performed in 47.2% (n = 17) while microscopic resection was performed in 52.8% (n = 19) patients. Within median follow-up of 18 months, no tumor recurrence or progress was seen. Female patients were more common in this study.

Endocrine outcome:

New hypopituitarism was defined as worsening of one or more pituitary axes after surgery or a new permanent arginine vasopressin deficiency persistent 3 months after surgery. Out of 36 patients with complete evaluation of pituitary function before and after surgery, three (8.3%) patients developed a new corticotrope insufficiency. New permanent arginine vasopressin deficiency (DI) was diagnosed in one patient (2.8%). All these patients were treated with a microscopic technique. The results are summarized in Table 2. Three (8.3%) patients showed improvements in hypopituitarism and corticotrope insufficiency (two patients after microscopic and one patient after endoscopic surgery). Functional adenomas were treated in 22.2% (n = 8) and all of them achieved biochemical remission. All but one were graded according to Knosp as 0–2. The distribution between surgical and microscopic resection was equal (n = 4, 50% in each cohort). We found no statistical difference between male and female patients in regard to endocrine outcome (*p* = 0.165). However, all patients with new pituitary insufficiency were female patients.

Extent of resection and intraoperative MRI:

GTR was achieved in 94.4% (n = 34) and we found no statistical difference between the surgical techniques (*p* = 0.935, Table 1). Additional resection due to tumor remnant found in iMRI was performed in three cases (8.3%, Table 1). This increased the number of GTRs from 88.8% (n = 32) to 94.4% (n = 34). In two cases, tumor remnant inside the cavernous sinus identified in iMRI was intentionally left behind. There was no significant difference between the surgical techniques (Table 1). Interestingly, the continued resection after iMRI due to potentially resectable adenoma remnant in all cases was in Knosp 3–4 adenomas.

Pituitary adenomas Knosp 0–2:

In the subgroup analysis of less invasive pituitary adenomas graded as Knosp 0–2, 2 (9.1%, 2/22) patients developed new corticotrope insufficiency after microscopic resection. We have found no new pituitary insufficiency in the endoscopic cohort. One patient (4.3%, N = 1/23) developed permanent arginine vasopressin deficiency after microscopic resection, while no permanent arginine vasopressin deficiency was found in the endoscopically treated cohort. All patients achieved GTR and in this cohort no adenoma remnant was found in iMRI or postoperative MRI. We have summarized the characteristics of Knosp subgroups in the Table 3.

**Figure 1:**
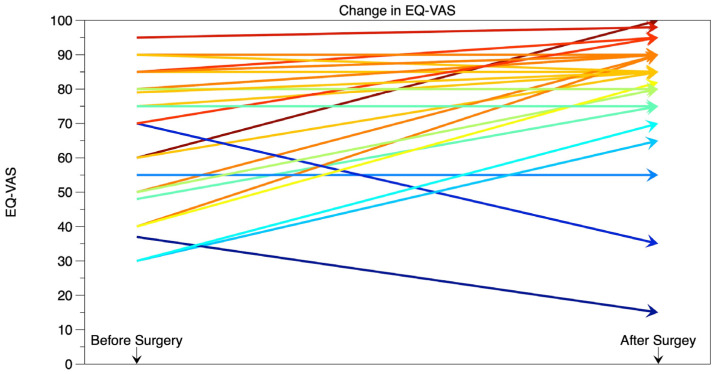
Results of EQ-VAS before and after transsphenoidal pituitary surgery. Different colors represent the reported EQ-VAS of different patients before and after surgery and its difference.

Quality of life:

Significant improvement in QoL was observed after the surgery, as indicated by the EQ-VAS scores (*p* = 0.003, Figure 1). The median EQ-VAS score increased from 70 before surgery to 85 after the surgery. When assessed using the EQ-5D3L, QoL either improved or remained unchanged in the majority of patients.

**Figure 2:**
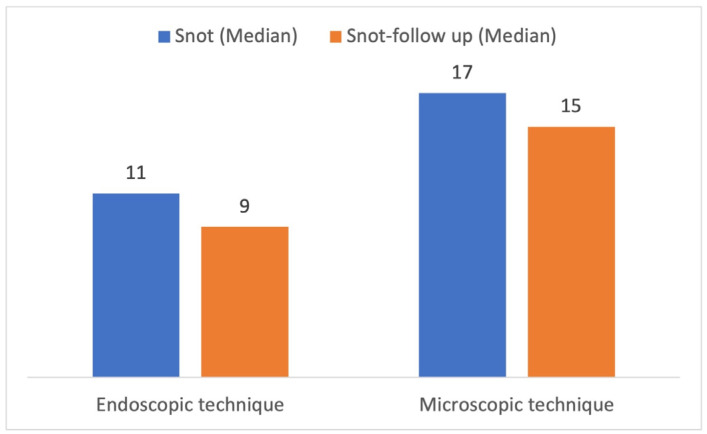
Comparison of results of the Sino-Nasal Outcome Test-20 scores for microscopic and endoscopic technique before (blue bar) and after (orange bar) surgery. Lower score means better quality of life.

Moreover, there was no significant difference in QoL between the endoscopic and microscopic surgery groups, as measured by both the EQ-VAS (*p* = 0.647) and the EQ-5D3L (*p* = 0.330). This suggests that both surgical approaches resulted in similar improvements in QoL for the patients. Furthermore, the SNOT-20 GAV analysis showed postoperative improvement 3 months after surgery (median 15.5 vs. 12). The results were independent on the surgical technique used (Figure 2). The detailed analysis is summarized in Table 4.

Visual function:

In 30.6% (n = 11) cases visual deficits were present before the surgery. All patients achieved improvement of visual function after surgery.

Complications:

No surgical complications, including revision surgery, epistaxis, meningitis, transient or permanent neurological deficits, or thromboembolic complications, were noted. Intraoperative CSF leak occurred in nine patients (25%) and was sealed with fat graft. No lumbar drains were used, and all patients were mobilized on POD 1. Microscopic technique was used in five and endoscope in four cases. No postoperative CSF rhinorrhea was noted.

## 4. Illustrative Case

**Figure 3:**
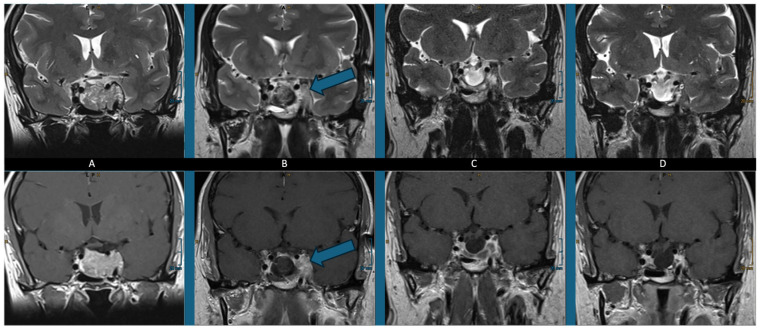
Demonstrative case of a 58-year-old woman with a nonfunctioning pituitary adenoma who presented with corticotropic insufficiency. Presented are T2 and contrast-enhanced T1 coronal sections of the sella. The preoperative MRI demonstrates a Knosp IV lesion with encased left ICA (**A**). The intraoperative MRI shows the resection cavity in the sella with a tumor remnant marked with the arrow (**B**). Further tumors could be removed after the intraoperative MRI leading to a gross total resection with no obvious remnant in the MRI after 3 months (**C**) and 1 year (**D**). No adjuvant treatment was administered.

To better demonstrate the intraoperative setup, we present a case of a 58 year old woman who presented with a corticotropic insufficiency. The MRI showed an intrasellar mass encasing the left ICA (Figure 3A). She was randomized to the endoscopic group. After a standard binostril approach, the opening of the sella was accomplished. Tumor resection was performed in the usual manner. The ICA on the left side was exposed and tumor within the cavernous sinus has been removed. Even with the angled endoscope, no obvious tumor could be detected, so iMRI was performed. Here, in the ventral part of the cavernous sinus, suspicious signal appeared (Figure 3B). After the scan was completed, the resection cavity was exposed again. With the help of the navigation system, the tumor remnant was identified and removed.

The patient had an unremarkable recovery and was discharged on 7th day after the surgery, as is usual in our department. Upon follow-up, the patient reported well-being, and MRI images showed still-marked restorative changes without evidence of tumor remnants (Figure 3C). One year after surgery, the patient reported their subjective well-being, EQ-VAS was 98 (95 before surgery), and in the MRI the restorative changes disappeared with still no hint of tumor recurrence (Figure 3D). One year after the surgery, the substitution therapy for the corticotropic insufficiency was not necessary anymore. Four years after adenoma resection, no recurrence was observed.

## 5. Discussion

We present a prospective and randomized single-blinded study comparing endoscopic and microscopic resection with the primary endpoint evaluating endocrine function. Unfortunately, due to insufficient recruiting, this study was terminated before the designed number of patients was reached. The main reason was the preference of patients for the endoscopic technique. Nevertheless, despite the low number of patients, all cases of new postoperative pituitary insufficiency or arginine vasopressin deficiency were diagnosed after the microscopic resection. This trend was first identified in retrospective studies and confirmed in the prospective but not randomized Transspher study, but a validation in a randomized prospective trial with the primary endpoint of endocrine function has not been published so far [10,15,16]. Even if we cannot generalize the results of our study based on above-described facts, our results go in line with these publications. In addition to our earlier publications showing this trend, especially the Transspher study, as the only prospective study comparing microscopic and endoscopic treatment, the benefit of endoscopic resection for pituitary function was underlined. Meta-analysis published by Chen et al. showed the advantage of the endoscopic technique with regard to arginine vasopressin deficiency as well as hypothyroidism [8]. Laws et al. suggested that endoscopic view might be more appropriate to preserve the endocrinological function due to better visualization and delineation of the pituitary gland [16]. Our experience confirms this trend. On the other hand, according to our results microscopic resection is still a safe and good option for the treatment of pituitary adenomas if endoscopy is not available.

As the secondary outcome, extent of resection was evaluated in our study. In our cohort, we achieved very high rate of GTRs, and in adenomas graded as Knosp 0–2, no tumor remnants were found with either technique. We only had one remnant inside the cavernous sinus in each arm, both in the subgroup of Knosp 3–4. Due to the small sample size, our results surely do not represent the whole spectrum of pituitary patients. Nevertheless, it shows a very good efficacy of this procedure in experienced hands. Furthermore, results are similar to the results of the Transspher study, which did not find a significant difference for extent of resection between microscopic and endoscopic surgery. iMRI is used routinely in our setting. Many studies have showed a potential benefit of iMRI in the resection of pituitary adenomas [17,18,19,20]. This study was not designed to answer the question of the value of iMRI in pituitary surgery. Additional resection of an adenoma remnant was carried out in 8.3% and all were graded as Knosp 3–4. Interestingly, this additional resection did not result in a new hypopituitarism. In the cohort of adenomas graded Knosp 0–2, no additional resection was necessary. These data support our earlier results, that iMRI might be even more beneficial for invasive adenomas. This should be viewed not only in terms of GTR but also of safety, since in complicated situations, unnecessary manipulation inside the cavernous sinus or opening of diaphragm can be avoided [20].

The improvement in the quality of life of patients undergoing transsphenoidal resection of pituitary adenoma is a crucial aspect of evaluating the effectiveness of this surgical intervention. Our findings demonstrate a significant enhancement in overall well-being among these patients. The EQ-VAS scores increased from a median of 70 before surgery to 85 after surgery. This notable improvement is consistent with similar studies in the literature, which have reported positive outcomes in overall quality of life measures following transsphenoidal resection of pituitary adenomas. 

Furthermore, our analysis of the EQ-5D3L questionnaire revealed either stability or improvement in these dimensions postoperatively, reinforcing the positive impact of the surgical procedure on various aspects of daily life. Similar trends have been observed in other studies, supporting the idea that transsphenoidal resection contributes to a comprehensive improvement in health-related quality of life [21,22]. These studies show an initial dip in the QoL early after surgery with a gradual improvement at 3 and 6 months after the procedure.

Furthermore, the SNOT-20 GAV score exhibited a noteworthy increase from 12 to 15.5 three months post-surgery. This positive trend suggests a beneficial impact on nasal and sinus symptoms. Interestingly, comparable studies in the literature have documented an initial deterioration of nasal symptoms within the first weeks after surgery, followed by a subsequent return to preoperative levels between 6 to 12 weeks later [23,24]. In accordance with our results, studies comparing endoscopic and microscopic techniques have failed to identify significant differences in sinonasal symptoms between the surgical techniques [25,26].

With respect to the timing of our QoL assessments following surgery, it is conceivable that we might observe additional enhancements in the estimated QoL during subsequent evaluations at later time points after surgery. Since our study was not designed to measure these additional parameters, from the published work, showing analogous findings, we can only assume this development.

In conclusion, our study adds to the existing body of evidence supporting the positive influence of transsphenoidal resection of pituitary adenoma on the quality of life of affected individuals independent of surgical technique [21,27]. The consistent improvement across various quality of life measures, as indicated by EQ-VAS, EQ-5D3L, and SNOT-20 GAV, highlights the multidimensional benefits of this surgical intervention. These findings are in line with prior research, emphasizing the importance of transsphenoidal resection in enhancing the overall health and well-being of patients with pituitary adenomas.

Considering the complication profile, our study shows that an experienced team within a neurosurgical center can probably reduce even very low risk profile of transsphenoidal surgery [28,29]. As discussed earlier, for sure because of the low number of patients in our study, we cannot draw definitive consequences from our results. Despite intraoperative CSF leak in 25% of patients, no case of a postoperative rhinoliquorrhea was observed. This aspect underlines the safe and effective application of fat graft [13].

Visual deficit avoidance and improvement are important goals of transsphenoidal pituitary surgery [3]. Our data support the notion that transsphenoidal resection of pituitary adenoma combined with indirect decompression has a very good prognosis for visual improvements [30]. Our results are congruent with the current literature [3,30,31].

*Limitations*

Unfortunately, due to inadequate recruiting, this study was stopped before the predefined number of patients could be included. Because of this, the planned statistical power could not be achieved, our results do not deliver the planned evidence, and we cannot make general conclusions. iMRI was performed at surgeon’s discretion in all cases, so it is not possible to rule out that the opportunity for additional resection after iMRI influenced the decision to scan and ultimately had an influence on the overall resection strategy. Since all pituitary surgeries at our center are scheduled for iMRI-assisted adenoma resection, we do not have any recent cohort of patients treated without iMRI for comparison.

## 6. Conclusions

The endoscopic technique seems to contribute to a better endocrine outcome, as well as a lower rate of new hypopituitarism and arginine vasopressin deficiency. Overall, in experienced hands, transsphenoidal adenomectomy results in an excellent outcome in terms of extent of resection, surgical complications, and QoL with both surgical techniques.

## Figures and Tables

**Table 1:** Patients and tumor characteristics.

	Total	Microsurgery	Endoscopy	*p*
n	36	52.8% (19)	47.2% (17)	
Age				
Mean	58.1 (SD 14.9)	59.8 (SD 15.3)	56.2 (SD 14.7)	0.49
Range	33–81	33–81	26–78	
Sex				
Female ratio	69.4% (25)	78.9% (15)	58.8% (10)	0.197
Median tumor Volume (cm^3^)	3.34	2.47	4.23	0.433
Range (cm^3^)	0.02–15.2	0.1–10.6	0.02–15.2	
Knosp 0–2	72.2% (26)	73.7% (14)	70.6% (12)	0.876
Knosp 3–4	27.8% (10)	26.3% (5)	29.4% (5)	
Adenoma subtype				0.594
Non-functioning	77.8% (28)	78.9% (15)	76.5% (13)	
GH	13.9% (5)	21.1% (4)	5.9% (1)	
ACTH	5.6% (2)	0	11.8% (2)	
Prolactinoma	2.8% (1)	0	5.9% (1)	
Gross total resection	94.4% (34)	94.7% (18)	94.1% (16)	0.936
Resection after iMRI	8.3% (3)	5.3% (1)	11.7% (2)	0.593

**Table 2:** Endocrine outcome after transsphenoidal adenomectomy.

	New Corticotrope Insufficiency	New Arginine-Vasopressin-Deficiency	No New Endocrine Deficits
n (36)	8.3% (3)	2.8% (1)	88.9% (32)
Microscopic technique	15.8% (3/19)	5.3% (1/19)	78.9% (15/19)
Endoscopic technique	0	0	100% (17/17)
Female ratio	100% (3/3)	100% (1/1)	65.6% (21/32)
Median tumor Volume (cm^3^)	2.89	1.00	3.34
Knosp 0–2	66.7% (2/3)	100% (1/1)	71.9% (23/32)
Knosp 3–4	33.3% (1/3)	0	28.1% (9/32)
Gross total resection	66.7% (2/3)	100% (1/1)	96.9% (31/32)
Resection after iMRI	0	0	9.4% (3/32)

**Table 3:** Detailed characteristics of Knosp 0–2 and Knosp 3–4 subgroups.

	Knosp 0–2	Knosp 3–4
n (36)	72.2% (26)	27.8% (10)
Microscopic technique	53.8% (14/26)	50% (5/10)
Endoscopic technique	46.2% (12/26)	50% (5/10)
Female ratio	73.1% (19/26)	60% (6/10)
Median tumor volume (cm^3^)	2.13	5.1
Gross total resection	100% (26/26)	80% (8/10)
Resection after iMRI	0	30% (3/10)

**Table 4:** Detailed characteristics of quality of life measurements.

	EQ-VAS Before Surgery	EQ-VAS After Surgery		EQ-5D-3L Improved in 1 or More Dimensions or Same as Before Surgery	Snot (Before Surgery)	Snot (After Surgery)
	70 (median)	85 (median)	*p* = 0.003	96.7% (N = 29/30)	15.5 median	12 (median)
Endoscopic technique	70 (median)	85 (median)		93.3% (N = 14/15)	11 (median)	8.5 (median)
Microscopic technique	70 (median)	85 (median)		100% (N = 15/15)	17 (median)	15 (median)
		*p* = 0.647		*p* = 0.330		

## Data Availability

Data available on demand by corresponding author.
